# Short-term functional outcome in psychotic patients: results of the Turku early psychosis study (TEPS)

**DOI:** 10.1186/s12888-021-03516-4

**Published:** 2021-12-02

**Authors:** Raimo K. R. Salokangas, Tiina From, Tuula Ilonen, Sinikka Luutonen, Markus Heinimaa, Reetta-Liina Armio, Heikki Laurikainen, Maija Walta, Janina Paju, Anna Toivonen, Päivi Jalo, Lauri Tuominen, Jarmo Hietala

**Affiliations:** 1 Department of Psychiatry, University of Turku, Kunnallissairaalantie 20, FIN-20700 Turku, Finland; 2 Department of Psychiatry, Turku University Hospital, Turku, Finland; 3 Royal Ottawa Mental Health Centre, Ottawa, Canada

**Keywords:** Functional outcome, Psychotic disorders, Clinical high risk of psychosis, Premorbid adjustment, Disorganization symptoms, Negative symptoms

## Abstract

**Background:**

Functional recovery of patients with clinical and subclinical psychosis is associated with clinical, neuropsychological and developmental factors. Less is known about how these factors predict functional outcomes in the same models. We investigated functional outcomes and their predictors in patients with first-episode psychosis (FEP) or a confirmed or nonconfirmed clinical high risk of psychosis (CHR-P vs. CHR-N).

**Methods:**

Altogether, 130 patients with FEP, 60 patients with CHR-P and 47 patients with CHR-N were recruited and extensively examined at baseline (T0) and 9 (T1) and 18 (T2) months later. Global Assessment of Functioning (GAF) at T0, T1 and T2 and psychotic, depression, and anxiety symptoms at T1 and T2 were assessed. Functional outcomes were predicted using multivariate repeated ANOVA.

**Results:**

During follow-up, the GAF score improved significantly in patients with FEP and CHR-P but not in patients with CHR-N. A single marital status, low basic education level, poor work situation, disorganization symptoms, perceptual deficits, and poor premorbid adjustment in patients with FEP, disorganization symptoms and poor premorbid adjustment in patients with CHR-P, and a low basic education level, poor work situation and general symptoms in patients with CHR-N predicted poor functional outcomes. Psychotic symptoms at T1 in patients with FEP and psychotic and depression symptoms at T1 and anxiety symptoms at T2 in patients with CHR-P were associated with poor functioning.

**Conclusions:**

In patients with FEP and CHR-P, poor premorbid adjustment and disorganization symptomatology are common predictors of the functional outcome, while a low education level and poor work situation predict worse functional outcomes in patients with FEP and CHR-N. Interventions aimed at improving the ability to work and study are most important in improving the functioning of patients with clinical or subclinical psychosis.

**Supplementary Information:**

The online version contains supplementary material available at 10.1186/s12888-021-03516-4.

## Background

Psychoses impose substantial human, medical, social and economic burdens on affected individuals, their families and society as a whole [1, 2] and are associated with a reduced life expectancy by 15–20 years [3]. Therefore, clarification of the factors affecting outcomes in patients with psychoses is of great importance.

The clinical outcomes of schizophrenia are still rather poor [4, 5], the need for care is high [6], and the ability to work is very low [7]. Compared to other psychotic disorders, schizophrenia is consistently characterized by poorer courses and outcomes [8]. Poor premorbid personal and psychosocial development, slow illness development, young age at the onset of illness, male gender, single marital status, lack of an interpersonal network, and alcohol and drug abuse are associated with poor outcomes, and among the clinical characteristics, severe negative symptoms, neurocognitive deficits and slow or partial recovery after the first illness episode are associated with poor outcomes [9–22].

In terms of neurocognitive deficits, the most pronounced impairments have been observed in processing speed and episodic memory [23–28]. However, during a long-term follow-up of patients with schizophrenia, baseline neurocognitive impairments did not correlate with the clinical outcome [29]. In addition, childhood adversities have been associated with psychotic disorders [30–33]. Less is known about their role in predicting the functional outcomes of patients with psychosis.

Studies of patients with a high risk of psychosis (CHR-P) have mainly focused on the transition to psychosis and its prevention [34–36]. Since these earlier studies, the rate of onset of psychoses among patients with CHR-P has decreased, and is currently at the level of approximately 20% at the two-year follow-up [37, 38]. According to a meta-analysis, both psychological and pharmacological interventions appear to reduce or delay conversion to psychosis but rarely improve functional outcomes in relation to the control conditions [39].

Patients with CHR-P are characterized by many clinical disorders, including impairments in work or educational functioning, social functioning and quality of life [40]. In addition to brief limited psychotic symptoms, e.g., positive symptoms, bizarre thinking, schizotypal personality disorder/features, depression, and disorganization, neurocognitive deficits and poor psychosocial adjustment have been identified as risk factors for the transition to psychosis in patients with CHR-P [41–47].

As shown in various studies, processing speed, deficits in motor speed, verbal memory, verbal learning, verbal fluency and executive function are associated with the onset of psychosis and poor functioning [47–54]. According to a systematic review, negative and disorganization symptoms and cognitive deficits predate frank psychotic symptoms, and are risk factors for poor functioning [55]. Additionally, patients with CHR-P often report high levels of childhood adversities [56, 57]. In follow-up studies, childhood adversities have predicted depression, poor social functioning and suicidal thinking [58, 59].

Premorbid psychosocial development and its role as a predictor of the functional outcome have received less attention. In a study of patients with CHR-P, premorbid psychosocial adjustment, baseline negative symptoms and poor working/schooling situations predicted poor functional outcomes during follow-up [60]. Additionally, in other studies, negative symptoms and impairments in role and social functioning have predicted poor functional outcomes at follow-up [52, 53, 61].

The literature described above indicates that a large number of individual factors are associated with functional outcomes in patients with clinical or subclinical psychotic symptoms. The effects of these factors probably overlap with each other. Less is known about which of them exerts an independent effect on patients’ functional outcomes.

### Aims of the study

In this study, which is part of the Turku Early Psychosis Study (TEPS), we aimed to describe our study samples and investigate 1) the short-term (up to approximately 18 months) functional outcomes of patients with first-episode psychosis (FEP) or confirmed (CHR-P) or nonconfirmed clinical high risk of psychosis (CHR-N) and 2) the factors predicting functional outcomes in these three patient groups. We hypothesized that if positive psychotic symptoms effect on follow-up functioning, functional outcome will be poorer in patients with FEP than in patients with CHR.

## Materials and methods

In the Supplementary Material, we have described the methods of the TEPS project in detail. Here, we concentrate on the methods of the TEPS functional outcome study comprising patients with FEP, CHR-P and CHR-N. The TEPS study program was carried out in accordance with the latest version of the Declaration of Helsinki. The study design and protocols were approved by the ethics committee of Turku University Hospital. Written informed consent was obtained from participants after the procedure had been fully explained to them.

### Participants

The study patients aged 18 to 50 years were recruited from mental hospitals (*N* = 183), psychiatric outpatient care units (*n* = 101) and primary care stations (*n* = 19) of the Turku University Hospital District in Finland between October 2011 and December 2017.

When an eligible patient attended psychiatric/primary care services for the first time, the personnel completed a screen, determining whether the patient was possibly psychotic or at high risk of psychosis. The TEPS study group assessed the completed screens and invited the patients who possibly fulfilled the inclusion criteria to participate in the study examinations. FEP was defined based on the Structured Clinical Interview for DSM-IV [62] criteria and included schizophrenia, delusional and bipolar psychoses, acute transient psychoses and other psychoses. CHR-P was defined based on the ultrahigh-risk criteria Attenuated Psychotic Symptoms, Brief Limited Psychotic Symptoms, and Genetic risk and reduction of function assessed with the 3.0/5.0 version of the Structured Interview for Prodromal Syndromes (SIPS/SOPS), including Global Assessment of Functioning (GAF) [63, 64]. The patients who were at high risk of psychosis according to the health-care personnel’s clinical impression but who did not fulfil the SIPS/SOPS criteria in the diagnostic examination were patients with a nonconfirmed high risk of psychosis (CHR-N). Exclusion criteria for study patients were a previous psychotic disorder and IQ < 70. The final TEPS sample comprises 130 patients with FEP, 60 patients with CHR-P, and 47 patients with CHR-N.

### Baseline examinations

At baseline, the study patients underwent extensive examinations (Supplementary Material). For this outcome study, in addition to socioeconomic background, patients’ premorbid adjustment (PAS) [65], Axis I diagnosis (SCID-I; DSM-IV) [62] and clinical high risk of psychosis (SIPS/SOPS, including GAF) [62, 63] were obtained in interviews. Additionally, patients completed questionnaires on childhood adverse and traumatic experiences (TADS) [66, 67] and social support or confidants (PSS-R) [68].

PAS evaluates difficulties in individuals’ premorbid adjustment (rated from 0 to 6 points) during four time domains: childhood (up to 11 years), early (12–15 years) and late adolescence (16–18 years) and adulthood (19+ years). Because not all participants had reached adulthood, only the childhood, early and late adolescence PAS domain scores were used in analyses.

In SIPS/SOPS, 5 positive, 6 negative, 4 disorganization and 4 general symptoms, schizotypal personality disorder and functioning (GAF), are assessed [62, 63]. In the present study, we used sums of positive, negative, disorganization and general symptoms as clinical predictors.

The TADS, which has been validated in a general population sample [67], produces information on five core domains: emotional abuse (EmoAb), physical abuse (PhyAb), sexual abuse (SexAb), emotional neglect (EmoNeg) and physical neglect (PhyNeg).

The social support questionnaire (PSS-R) [68] comprises lists of 12 questions on support received (0 = never, 1 = hardly ever, 2 = sometimes, 3 = most of time and 4 = all the time) from family and friends. The sum score (range 0–48) was used as an indicator of confidant support.

Neuropsychological tests were not performed before the patients with psychosis recovered from their manifest psychosis, usually 3 to 4 weeks after admission to a hospital ward. The following domains and tests were used: estimate of premorbid cognitive functioning (Vocabulary, WAIS-III) [69], letter (S) and category (animals) fluency [70, 71], attention/vigilance (Trail Making A/B) [72], speed of processing (Digit Symbol, WAIS-III), verbal and visual working memory (Letter-Number Span, WAIS-III and Spatial Span WMS-R) [73], verbal learning (Hopkins Verbal Learning Test-Revised, HVLT) [74], visual learning (Brief Visuospatial Memory test-Revised, BVMT-R) [75], reasoning and problem solving (Neuropsychological Assessment Battery, Mazes) [76], perceiving and forming concepts in a complex problem solving situation (Rorschach Comprehensive System, RCS) [77, 78], executive function (Wisconsin Card Sorting Test, WCST) [79], and social cognition (Mayer-Salovey-Caruso Emotional Intelligence Test, MSCEIT, Managing Emotions, D and H) [80].

### Follow-up examinations

When nine (T1) and 18 (T2) months was elapsed from the baseline examination, patients were invited for extensive follow-up examinations (Supplementary Material). For this outcome study, GAF, as well as the occurrence of psychotic (yes/no), depression (yes/no) and anxiety (yes/no) symptoms were recorded at the follow-up time points (T1 and T2), and the transition to psychosis was detected [43]. Due to drop-outs, information regarding both functioning (GAF) and psychiatric symptoms at follow-up visits was supplemented by scrutinizing patients’ medical case notes and by telephone interviews of patients and/or their relatives and/or their doctors (RKRS), as described in detail in the Supplementary Material. Symptom severity was not assessed.

### Statistical analyses

First, distributions of background factors were cross-tabulated, and means (SD) of GAF, SIPS, TADS, confidant support, PAS scores and neuropsychological test scores were calculated by diagnostic groups and tested using the Chi-square test and ANOVA. Repeated measures ANOVA over the whole study period (GAFT0, GAFT1 and GAFT2) was calculated for the diagnostic group and each baseline characteristic, SIPS and TADS domain, three PAS domain scores and for each neuropsychological test.

Using multivariate repeated-measures ANOVAs, functioning over the study period (GAFT0, GAFT1 and GAFT2) was explained by blocks of independent variables: 1) diagnosis and background factors, 2) SIPS domain scores (positive, negative, disorganization and general) and confidant support, 3) neuropsychological test scores, 4) TADS domains and 5) PAS domain scores. At each stage, non-significant (*p* > 0.1) factors were omitted, and the remaining variables were included in the model with the next block of variables. In the final model, only the factors with a significant association (*p* < 0.05) with functioning were included in the equation. Thereafter, psychotic, depression and anxiety symptoms at T1 and T2 were entered into the final model to analyse how follow-up symptoms change the explanatory power of the model. Analyses were performed for all study subjects and separately for patients with FEP, CHR-P and CHR-N. Data were analysed using Statistical Program for the Social Sciences (SPSS) v24.0, and *p*-values < 0.05 were considered significant.

## Results

### Baseline sociodemographic and clinical characteristics

In terms of sociodemographic factors and social support received, no differences were observed between patients with FEP, CHR-P and CHR-N. One-third of patients with FEP had affective psychosis, and two-thirds had non-affective psychosis. The majority of the patients with CHR-P and CHR-N suffered from depressive and anxiety disorders; the difference was not significant (*p* = 0.369) (Table 1). SIPS psychotic symptoms, which were used as diagnostic criteria for FEP, CHR-P and CHR-N, increased linearly from CHR-N to CHR-P (*p* < 0.001) and from CHR-P to FEP (p < 0.001). Differences in disorganization symptoms between patients with FEP and CHR-N (*p* = 0.001) and between patients with CHR-P and CHR-N (*p* = 0.046) were also significant. In terms of negative and generalized symptoms, TADS domains and PAS domain scores, no significant differences were observed between diagnostic groups (Table 1).

**Table 1:** Socio-demographic background and baseline characteristics of the TEPS sample

	FEP^a^	CHR-P^b^	CHR-N^c^	All	*p*
**Gender (%)**	*n* = 130	*n* = 60	*n* = 47	*n* = 237	0.573
Male	56.9	53.3	55.8	55.8	
Female	43.1	46.7	44.2	44.2	
Age (years)	*n* = 130	*n* = 60	*n* = 47	*n* = 237	0.289
18–23	37.7	53.3	46.8	43.5	
24–29	33.1	23.3	23.4	28.7	
30–49	29.2	23.3	29.8	27.8	
mean (SD)	26.5(5.9)	25.0(6.2)	27.1(7.9)	26.3(6.4)	0.195
**Marital status (%)**	*n* = 130	*n* = 60	*n* = 47	*n* = 237	0.536
Single	72.3	71.7	63.8	70.5	
Ever married/divorced	27.7	28.3	36.2	29.5	
Living with	*n* = 130	*n* = 60	*n* = 47	*n* = 237	0.960
Alone	47.7	45.0	46.8	46.8	
Own family/children	30.0	30.0	31.9	30.4	
Parents/sisters	16.2	20.0	12.8	16.5	
Other persons	6.2	5.0	8.5	6.3	
**Basic education (%)**	*n* = 130	*n* = 60	*n* = 47	*n* = 237	0.490
Comprehensive school or less	40.0	41.7	44.7	41.4	
High school	13.8	6.7	6.4	10.5	
College	46.2	51.7	48.9	48.1	
**Professional education (%)**	*n* = 130	*n* = 60	*n* = 47	*n* = 237	0.797
None	43.8	48.3	42.6	44.7	
Vocational school	41.5	43.3	42.6	42.2	
University	14.6	8.3	14.9	13.1	
Years of education; mean (SD)	13.8(3.1)	13.2(2.9)	13.9(3.4)	13.7(3.1)	0.382
**Work situation (%)**	*n* = 130	*n* = 60	*n* = 47	*n* = 237	0.574
Employed	60.0	58.3	61.7	59.9	
Unemployed	19.2	30.0	21.3	22.4	
Sick leave	10.8	6.7	6.4	8.9	
Temporary retirement	10.0	5.0	10.6	8.9	
**Confident support (score)**	*n* = 104	*n* = 48	*n* = 42	*n* = 194	
mean, SD	31.4(11.5)	27.4(10.7)	29.7(7.7)	30.0(10.7)	0.102
**SCID Diagnosis (%)**	*n* = 130	*n* = 60	*n* = 47	*n* = 237	< 0.001
No	0.0	8.3	6.4	3.4	
Bipolar	16.9	3.3	8.5	11.8	
Depression	16.9	56.7	63.8	36.3	
Nonaffective Psychosis	66.2	5.0	0.0	37.6	
Anxiety	0.0	26.7	21.3	11.0	
**SIPS symptom score mean (SD)**	*n* = 125	*n* = 60	*n* = 47	*n* = 232	
Positive (0–30)	16.5(5.3)	10.6(5.9)	5.9(4.2)	12.8(6.4)	< 0.001
Negative (0–30)	11.0(7.3)	11.4(6.6)	10.2(6.1)	10.9(6.8)	0.645
Disorganized (0–24)	5.4(4.1)	4.8(3.5)	3.4(2.3)	4.9(3.7)	0.006
General (0–24)	6.8(4.6)	7.7(3.3)	6.3(3.8)	6.9(4.1)	0.166
**TADS score mean (SD)**	*n* = 107	*n* = 48	*n* = 40	*n* = 195	
EmoAb (1–5)	4.7(4.3)	5.3(4.4)	5.4(4.8)	5.0(4.4)	0.586
PhyAb (1–5)	1.7(2.1)	1.8(2.6)	2.3(3.9)	1.9(2.7)	0.508
SexAb (1–5)	1.2(3.1)	0.6(1.6)	1.2(3.9)	1.1(3.0)	0.520
EmoNeg (1–5)	6.8(5.0)	7.5(5.1)	8.3(4.1)	7.3(4.9)	0.241
PhyNeg (1–5)	3.4(2.8)	3.3(3.1)	4.4(3.5)	3.5(3.1)	0.156
**PAS score mean (SD)**	*n* = 124	*n* = 57	*n* = 47	*n* = 228	
-11 years (0–24)	6.2(3.6)	6.8(3.3)	6.6(3.6)	6.5(3.5)	0.495
12–15 years (0–30)	10.1(5.2)	10.8(4.9)	10.0(4.1)	10.3(4.9)	0.669
16–18 years (0–30)	10.9(5.6)	10.9(5.9)	10.4(3.8)	10.8(5.3)	0.839

^a^First-episode psychosis

^b^Confirmed clinical high-risk to psychosis

^c^Nonconfirmed clinical high risk to psychosis

*EmoAb* Emotional abuse, *PhyAb* Physical abuse, *SexAb* Sexual abuse

*EmoNeg* Emotional neglect, *PhyAb* Physical Abuse, *PAS* Premorbid adjustment

The use of mental services and the duration of symptoms were investigated to describe patients’ recent clinical history. Before baseline admission or outpatient visits, 65.4% of patients with FEP, 76.7% of patients with CHR-P and 85.1% of patients with CHR-N sought help for their mental problems (*p* = 0.024). At baseline, 44.4% of patients reported anxiety, and 40.1% reported depression symptoms (*p* > 0.1). In patients with FEP, delusional (41.5%), hallucinatory (37.7%) and disorganization symptoms (33.8%) were more prevalent (ANOVA: *p* = 0.013, *p* = 0.001, and *p* = 0.011, respectively) than in patients with CHR-P (23.3, 13.3, and 18.3%, respectively) and patients with CHR-N (23.4, 19.1, and 14.9%, respectively), including subclinical symptoms. The mean duration of symptoms was 555 (SD 1302) days for anxiety, 337 (SD 649) days for depression, 181 (SD 691) days for delusions, 88 (SD 279) days for hallucinations and 197 (SD 770) days for disorganization. No differences were observed between diagnostic groups. Alcohol was related to the onset of symptoms in 6.8% of patients and substance use was related in 5.5% of patients, but no differences were observed between patient groups. The FEP sample included three patients with substance-induced psychoses.

In general, patients with FEP had more difficulties in maintaining attention, processing speed, working memory, verbal and visual learning, reasoning and problem solving than patients with CHR-P or CHR-N; differences between patients with CHR-P and CHR-N were nonsignificant (Table 2). In WCST and RCS, patients with FEP exhibited poorer performance than patients with CHR-N, and in RCS, patients with CHR-P patients exhibited poorer performance than patients with CHR-N (Table 2).

**Table 2:** Neuropsychological tests by diagnostic groups

	FEP^a^ (*n* = 119)		CHR-P^b^ (*n* = 54)		CHR-N^c^ (*n* = 46)		All (*n* = 219)			FEP/CHR-P	FEP/CHR-N	CHR-P/CHR-N
	Mean	SD	Mean	SD	Mean	SD	Mean	SD	p1	p2	p3	p4
Verbal skills												
WAIS III Vocabulary	42.61	10.84	42.80	10.57	43.07	10.85	50.14	7.54	0.970	0.914	0.806	0.901
MCCB Category Fluency	22.23	5.71	22.98	5.24	24.52	7.39	24.36	6.12	0.090	0.444	**0.029**	0.202
MCCB S-words	15.11	5.56	15.54	5.48	16.83	4.45	16.51	5.40	0.180	0.625	0.065	0.229
Attention/vigilance												
TMT-A	32.94	10.18	28.17	8.33	31.52	10.93	29.70	9.83	**0.015**	**0.004**	0.411	0.093
TMT-B	92.18	66.00	86.24	82.34	66.50	38.08	75.81	58.28	0.082	0.583	**0.026**	0.137
Speed of processing												
WAIS III Digit Symbol	66.37	15.78	73.44	16.85	74.59	15.54	75.24	16.97	**0.002**	**0.008**	**0.003**	0.722
Working memory												
WAIS III Letter-Number Span	9.97	2.22	10.59	2.02	11.17	2.34	10.94	2.38	**0.006**	0.088	**0.002**	0.189
WMS-R Spatial Span	16.87	3.01	17.57	2.95	17.22	2.15	17.63	2.88	0.313	0.126	0.477	0.523
Verbal learning												
HVLT immediate	25.94	4.29	27.31	4.60	28.89	3.95	27.79	4.43	**< 0.001**	0.053	**< 0.001**	0.069
HVLT delayed	9.18	2.12	10.19	1.90	10.35	1.72	10.04	1.95	**< 0.001**	**0.002**	**0.001**	0.684
Visual learning												
MCCB BVMT-R	24.37	6.98	26.96	6.43	26.65	6.51	26.87	6.59	**0.029**	**0.020**	0.053	0.819
Reasoning and problem solving												
MCCB NAB Mazes	20.43	4.89	22.07	3.59	22.24	4.15	21.78	4.18	**0.018**	**0.025**	**0.020**	0.854
WCST												
Total number of errors	30.20	24.54	28.24	19.23	22.35	18.18	28.07	22.22	0.125	0.589	**0.042**	0.185
Perseverative responses	18.73	19.06	16.63	12.98	12.91	11.21	16.99	16.39	0.122	0.433	**0.041**	0.257
Perseverative errors	16.36	15.00	15.11	10.91	11.87	9.47	15.11	13.12	0.143	0.560	**0.049**	0.218
Percent conceptual level responses	64.51	21.74	66.07	16.94	71.46	17.17	66.36	19.84	0.130	0.630	**0.044**	0.176
Number of categories completed	4.96	1.68	5.11	1.42	5.39	1.31	5.09	1.55	0.272	0.547	0.108	0.368
Ro-Exner (RCS)												
Perceptual thinking index (PTI)	1.25	1.34	1.37	1.19	0.70	1.07	1.16	1.27	**0.016**	0.566	**0.011**	**0.008**
Distorted perception (X-%)	0.28	0.11	0.28	0.09	0.21	0.08	0.26	0.10	**0.001**	0.824	**0.001**	**0.002**
Incoherent thinking (WSum6)	9.90	8.70	9.69	7.56	7.02	6.50	9.24	8.06	0.108	0.871	**0.040**	0.099
Appropriate answers (XA%)	0.70	0.12	0.70	0.09	0.76	0.09	0.71	0.11	**0.002**	0.923	**0.001**	**0.003**
Extended appropriate answers (WDA%)	0.73	0.12	0.73	0.09	0.79	0.08	0.75	0.11	**0.003**	0.902	**0.001**	**0.005**

^a^First episode psychosis

^b^Confirmed clinical high risk to psychosis

^c^Nonconfirmed clinical high risk to psychosis; p1 = ANOVA for all patients; p2 = ANOVA for FEP/CHR-P; p3 = ANOVA for FEP/CHR-N; p4 = ANOVA for CHR-P/CHR-N

### Baseline drug treatment

The study patients did not participate in any specific trial intervention; their treatment followed usual clinical practice, including supportive therapy and drug treatment. By the time of baseline examination, patients with FEP had received neuroleptic drugs (87.7%) more often (*p* < 0.001) and antidepressants (47.7%) less often (*p* = 0.001) than patients with CHR-P (45.0 and 48.3%) and CHR-N (31.9 and 53.2%). Notably, 20.3% of all patients received anxiolytics, 9.3% received hypnotics and 6.3% were treated with mood stabilizers, with no differences between diagnostic groups.

### Functioning at baseline and follow-ups

A comparison of patients’ functioning, as assessed using the Global Assessment of Functioning (GAF), between diagnostic groups showed that at baseline, the functioning of patients with FEP was poorer than that of patients with CHR-P (*p* = 0.004) and CHR-N (*p* < 0.001). The difference between patients with CHR-P and CHR-N was not significant (*p* = 0.072). During follow-up, the functioning of patients with FEP and CHR-P improved significantly, but not that of patients with CHR-N (Table 3). At T1 and T2, no significant differences in functioning were observed between diagnostic groups (Table 3).

**Table 3:** Patients’ functioning at baseline (T0), at 9 months (T1) and at 18 months follow-ups (T2) (A) and paired differences for functioning at baseline (GAFT0), at 9 months (GAFT1) and at 18 months follow-ups (GAFT2) (B) by diagnostic groups

	FEP^a^			CHR-P^b^			CHR-N^c^			ALL			p1		
	T0	T1	T2	T0	T1	T2	T0	T1	T2	T0	T1	T2	T0	T1	T2
	*n* = 130	*n* = 130	*n* = 129	*n* = 60	*n* = 59	*n* = 59	*n* = 47	*n* = 47	*n =* 47	*n* = 237	*n* = 236	*n* = 235			
A															
GAF (%)															
Good (61–100)	15.4	37.7	43.4	18.3	32.2	44.1	25.5	42.6	48.9	18.1	37.3	44.7	< 0.001	0.053	0.053
Moderate (41–60)	46.2	33.8	29.5	66.7	54.2	45.8	70.2	38.3	34.0	56.1	39.8	34.5			
Poor (0–40)	38.5	28.5	27.1	15.0	13.6	10.2	4.3	19.1	17.0	25.7	22.9	20.9			
Mean	48.0	57.1	59.3	53.7	56.6	62.2	58.2	59.4	61.6	51.4	57.4	60.5	< 0.001	0.679	0.548
SD	14.4	18.7	19.7	10.8	15.1	17.2	9.1	16.2	17.4	13.2	17.3	18.6			
B															
	Mean	SD		t	p										
FEP^a^ (df = 128–129)															
GAFT0 - GAFT1	−9.18	17.75		−5.90	< 0.001										
GAFT0 - GAFT2	−11.48	18.95		−6.88	< 0.001										
GAFT1 - GAFT2	−2.31	14.00		−1.87	0.063										
CHR-P^b^ (df = 58)															
GAFT0 - GAFT1	−2.31	13.61		−1.30	0.198										
GAFT0 - GAFT2	−7.88	17.84		−3.39	0.001										
GAFT1 - GAFT2	−5.58	15.07		−2.84	0.006										
CHR-N^c^ (df = 46)															
GAFT0 - GAFT1	−1.23	16.86		−0.50	0.618										
GAFT0 - GAFT2	−3.47	16.43		−1.45	0.155										
GAFT1 - GAFT2	−2.23	8.31		−1.84	0.072										
ALL (df = 234–235)															
GAFT0 - GAFT1	−5.88	16.96		−5.32	< 0.001										
GAFT0 - GAFT2	−8.97	18.39		−7.48	< 0.001										
GAFT1 - GAFT2	−3.11	13.38		−3.57	< 0.001										

^a^First-episode psychosis

^b^Confirmed clinical high-risk to psychosis

^c^Nonconfirmed clinical high risk to psychosis. p1 = significance between FEP, CHR-P and CHR-N

### Univariate prediction of functioning

First, repeated-measures ANOVA separately predicted follow-up functional outcomes (GAFT0, GAFT1 and GAFT2) with three sets of variables: 1. diagnostic groups and baseline sociodemographic factors, 2. SIPS symptoms, TADS domains and PAS domains, and 3. neuropsychological test scores. The diagnostic groups (FEP/CHR-P/CHR-N) (*p* = 0.064) and SCID diagnosis groups (*p* = 0.140) were not significantly associated with the functional outcome. Among the baseline characteristics, female gender (*p* = 0.001), nonsingle marital status (*p* = 0.005), confidant support (*p* = 0.005), good basic (*p* < 0.001) and professional education (p < 0.001) and a good work situation (p < 0.001) predicted good functioning (Table 4). In patients with FEP, these associations were similar to those in the entire patient sample. Only gender and work situation in patients with CHR-P and basic and professional education and the work situation in patients with CHR-N were associated with functioning at follow-up (Supplementary Table 1).

**Table 4:** Repeated measures ANOVA of functional outcome for all patients (*n* = 213)

Between subjects effect	df	F	p	Par. η^2^	
Basic education	2	6.22	**0.002**	0.06	
Work situation	3	9.29	**< 0.001**	0.12	
SIPS disorganized symptoms	1	24.62	**< 0.001**	0.11	
Appropriate answers (XA%)	1	4.14	**0.043**	0.02	
Late adolescence PAS	1	18.29	**< 0.001**	0.08	
Parameter estimations	B	t	p	CI95%	
Functioning at baseline (GAFT0)					
Comprehensive school or less	0.81	0.49	0.627	−2.47	4.09
High school	−2.73	−1.09	0.277	−7.69	2.22
College	–				
Employed	9.60	3.53	**0.001**	4.24	14.96
Unemployed	8.31	2.78	**0.006**	2.42	14.21
Sick leave	3.42	0.96	0.337	−3.58	10.42
Temporary retirement	–				
SIPS disorganized symptoms	−1.30	−5.81	**< 0.001**	− 1.75	−0.86
Appropriate answers (XA%)	20.32	2.85	**0.005**	6.24	34.40
Late adolescence PAS	− 0.23	−1.48	0.140	− 0.55	0.08
Functioning at 9 months (GAFT1)					
Comprehensive school or less	−4.44	−2.05	0.042	−8.72	−0.17
High school	−9.31	−2.85	**0.005**	−15.76	−2.86
College	–				
Employed	11.89	3.36	**0.001**	4.90	18.87
Unemployed	5.13	1.32	0.189	−2.55	12.81
Sick leave	6.23	1.35	0.180	−2.89	15.34
Temporary retirement	–				
SIPS disorganized symptoms	−1.01	−3.46	**0.001**	−1.59	−0.44
Appropriate answers (XA%)	14.65	1.58	0.117	−3.68	32.99
Late adolescence PAS	−0.75	−3.64	**< 0.001**	−1.15	− 0.34
Functioning at 18 months (GAFT2)					
Comprehensive school or less	−5.99	−2.52	**0.012**	−10.67	−1.31
High school	−11.62	−3.24	**0.001**	−18.68	−4.55
College	–				
Employed	14.37	3.71	**< 0.001**	6.72	22.01
Unemployed	5.42	1.27	0.205	−2.99	13.82
Sick leave	8.80	1.74	0.083	−1.18	18.79
Temporary retirement	–				
SIPS disorganized symptoms	−0.81	−2.53	**0.012**	−1.44	−0.18
Appropriate answers (XA%)	5.79	0.57	0.570	−14.28	25.87
Late adolescence PAS	−0.91	−4.05	**< 0.001**	−1.36	−0.47

Par. η^2^ = Partial Eta Squared. Significant p values **bolded**

All SIPS symptoms, PAS domains and PhyAb from TADS were significantly associated with functioning (Supplementary Table 2). In patients with FEP, all SIPS symptoms and all PAS domains, in patients with CHR-P, SIPS negative and disorganized symptoms, PhyAb and all PAS domains and in patients with CHR-N, all SIPS symptoms, EmoAb and PhyAb associated significantly with follow-up functioning. In patients with FEP and CHR-P, but not in patients with CHR-N, both childhood, early and late adolescence PAS scores were associated with follow-up functioning (Supplementary Table 2).

From neuropsychological tests, WAIS IQ (vocabulary), word fluency, divided attention (TMTB), processing speed, verbal and visual working memory, verbal and visual learning and problem solving were associated with functioning. From the WCST, the number of errors, conceptual responses and from RCS test perceptual problems (PTI, X-%, XA%, and WDA%) associated with functioning. Vocabulary, word fluency, visual working memory, visual learning, and perceptual disturbances in patients with FEP; sustained attention (TMTA), verbal working memory and WCST scores in patients with CHR-P; and vocabulary and attention (TMTA) in patients with CHR-N were associated with functioning (Supplementary Table 3).

### Multivariate prediction of functioning at follow-up

Next, the functional outcome (GAFT0, GAFT1 and GAFT2) was predicted by all variables in multivariate repeated ANOVAs. Predictors were added to the models in order as described in the Methods. In the entire patient sample, a good basic education and work situation, lack of disorganization symptoms and perceptual deficits, and good premorbid late adolescence adjustment predicted good functioning. Together, these factors explained 38.8% of the variance in functioning (Table 4). When the baseline drugs were added into the model (Table 4), only neuroleptics associated negatively with the functional outcome (*p* = 0.003, η^2^ = 0.042) and the model did not change essentially. The effect of perceptual deficits was not anymore significant (*p* = 0.071, η^2^ = 0.016).

In sensitivity analyses of patients with FEP, marital status (*p* = 0.001), basic education (*p* = 0.012), work situation (*p* = 0.002), SIPS disorganization symptoms (*p* < 0.001), perceptual problems (XA%; *p* = 0.028) and late adolescence PAS scores (*p* = 0.003) were significantly associated with functioning (Supplementary Table 4a). In patients with CHR-P, only baseline SIPS disorganization symptoms (*p* = 0.008) and childhood PAS scores (*p* = 0.004) were associated with functioning (Supplementary Table 4b), whereas in patients with CHR-N, basic education (*p* = 0.001), work situation (*p* = 0.009) and SIPS general symptoms (*p* = 0.006) were associated with functioning (Supplementary Table 4c). The proportion of variance explained was 55.6% for patients with FEP, 24.2% for patients with CHR-P, and 72.5% for patients with CHR-N. In the patients with FEP, baseline medication did not associate with the functional outcome. In the patients with CHR-P, baseline anxiolytics associated negatively with the functional outcome (*p* = 0.016, η^2^ = 0.104) and the effect of late adolescence PAS became insignificant (*p* = 0.053, η^2^ = 0.069). In the patients with CHR-N, baseline medication had no significant association with the functional outcome.

In the final stage, psychotic, depressive and anxiety symptoms occurring at T1 and T2 were included in the outcome models. In the entire patient sample, T1 psychotic symptoms (*p* < 0.001), T2 depression (*p* = 0.001) and T2 anxiety symptoms (*p* = 0.006) were entered into the equation, and the proportion of variance explained increased from 38.8 to 63.3%. Only T1 psychotic symptoms (*p* < 0.001) in patients with FEP; T1 psychotic symptoms (*p* = 0.023), T1 depression (*p* = 0.002) and T2 anxiety symptoms (*p* < 0.001) in patients with CHR-P; and T2 depression (*p* = 0.046) symptoms in patients with CHR-N were entered into the equation. The proportion of variance in functioning explained increased in patients with FEP (from 55.6 to 73.8%) and CHR-P (from 24.2 to 90.0%), but not in patients with CHR-N (from 72.5 to 68.5%). Transitions to psychosis (CHR-P: 13/60 [21.7%]; CHR-N: 6/47 [12.8%]) were not associated with functional outcomes in either CHR group.

## Discussion

At the time of a visit to receive treatment, patients with FEP, CHR-P or CHR-N did not differ in their sociodemographic background, childhood adverse experiences or premorbid adjustment. In terms of premorbid functioning, subjects with CHR-P did not differ from patients with first-episode psychosis or multiepisode schizophrenia [49].

At baseline, the functioning of patients with FEP was poorer than that of patients with CHR-P and CHR-N, but this difference became equalized during follow-up; functioning improved in patients with FEP and CHR-P, but not in patients with CHR-N. In patients with FEP, resolving psychotic symptoms were associated with reduced functioning in the acute phase (Supplementary Table 2) and possibly explained their good functional recovery. In patients with CHR-P, the recovery of functioning was slower and was not related to psychotic symptoms. Instead, negative and disorganization symptoms were significantly associated with functioning at baseline and at the 9-month follow-up visit, but not at the 18-month follow-up (Supplementary Table 2), indicating that improvements in negative and disorganization symptoms were possibly related to improvements in functional outcomes. The interventions possibly focused on reducing positive psychotic symptoms did not considerably improve functioning in patients with CHR-N.

At the eighteen-months follow-up, 43.4% of patients with FEP functioned well, 29.5% functioned moderately, and 27.1% functioned poorly. The proportion of patients with good functioning was slightly higher than that reported in previous studies [81, 82]. Remarkably, the sociodemographic background, SIPS symptoms, neurocognitive deficits and premorbid adjustment were extensively associated with functioning. However, in the multivariate model, only basic education (high), work situation (good), disorganization symptoms (few), perceptual disturbances (few) and late adolescence premorbid adjustment (good) predicted significantly good functional outcomes. However, constructions of predictive factors for functional outcome varied considerably between patients with FEP, CHR-P and CHR-N.

In patients with FEP, the marital status (single), poor basic education and poor work situation were strongly associated with a poor functional outcome. Marital status, education and work situation, central components of social competence [83, 84] represent endpoints of psychosocial development at the beginning of the first visit for treatment. Interestingly, these three sociodemographic factors were still powerful predictors, although the effect of adolescent development [64] was considered. Possibly since adolescence, psychiatric symptomatology and neuropsychological deficits with difficulties in schooling shift both sociosexual and work performance development to the low competence trajectory with further worsening after the onset of psychosis. In patients with chronic schizophrenia, extremely low competence was observed in single men whose quality of life was exceptionally low [85].

In patients with CHR-P and CHR-N, the roles of education and work situation varied, as the latter was a significant predictor, but not the former. In our previous studies on patients with CHR-P [60, 86], the work situation and education predicted functioning at the short-term follow-up. In the present study, the small number of patients with CHR-P may explain the difference; in a combined sample, both education and the work situation were significant predictors (analyses available by request), indicating that they are both important predictors of functioning in patients with subclinical psychotic symptoms.

Among the baseline symptoms, only SIPS disorganization symptoms displayed power in independently predicting functional outcomes of both patients with FEP and CHR-P. The effect of negative symptoms on functioning, which has been reported in several studies on patients with schizophrenia [15, 17, 18, 20, 22] and in bivariate analyses of the present study, was replaced by the effects of work situation and disorganization symptoms. Positive symptoms correlated with baseline functioning, but in the model, they did not significantly predict functioning at follow-up. In a follow-up study of patients with FEP, remission of negative symptoms was a critical predictor of future functioning [81]. Consistent with earlier studies [20, 22, 60, 87], the prevention of major psychotic symptoms, delusions and hallucinations, hardly exert a substantial effect on the functional outcomes of patients with psychosis. In patients with CHR-P, the role of positive symptoms is similar. Similar to our previous studies [60, 86] examining patients with CHR-P, positive symptoms and the transition to psychosis were not associated with functional outcomes. Positive psychotic symptoms (such as delusions and hallucinations) are specific predictors of new or incident psychotic disorders [88–90], but they rarely play any role in predicting functional outcomes of patients with CHR-P.

In bivariate analyses, various neurocognitive deficit indicators were associated with functional outcomes, but in the multivariate model, only perceptual disturbances, as assessed using the Rorschach Exner (RCS) test, were specifically independently associated with poor functional outcomes in patients with FEP. This type of disturbance in visual form perceptions differs from sensory disturbances, such as hallucinations. Interestingly, deficits in visual learning (BVMT-R) were also associated with poor functional outcomes, but in model, they lost their predictive power when premorbid adjustment was entered into the model. Perceptual disturbances, possibly caused by the incoherent structure and function of the CNS neuronal network, appear to play an important role in functional outcomes among patients with psychosis. In patients with CHR-P and CHR-N, neurocognitive deficits played a minor role in predicting the functional outcome. Thus, although neurocognitive deficits are common in patients with FEP [23–28] and CHR-P [47–54] and correlate with actual functioning, their value in predicting functional outcomes is not great.

Consistent with earlier studies [14–22, 60], premorbid adjustment, assessed using the PAS scale, was strongly associated with functional outcomes in patients with FEP and CHR-P. In both patient groups, late adolescence PAS scores had the strongest association with functioning at follow-up. The first signs of schizophrenia often occur in adolescence. While the PAS assessment represents a global assessment of individuals’ early psychosocial development, it also includes the effects of genetic and environmental factors, such as childhood adversities.

Remarkably, although childhood adversities were associated with the onset of psychotic and subpsychotic disorders, such as CHR [30–33, 56, 57], and with the transition to psychosis in patients with CHR [91], they did not predict functioning at follow-up in the multivariate model used in the present study.

Similar to other studies on patients with CHR-P [45, 92], affective disorders, both depression and anxiety were very common at the time the patients sought help. During follow-up, the transition to psychosis was not associated with functioning, and the association of positive psychotic symptoms was slight, while depressive and anxiety symptoms were strongly associated with poorer functioning and greatly increased the predictive power of the model. Based on these findings, affective and not positive psychotic symptomatology is the major psychiatric symptomatology worsening functional outcomes in patients with CHR-P. Additionally, in other studies of patients with CHR-P, a nonpsychotic comorbidity was associated with poor functional outcomes [93, 94].

A comparison of short-term functional outcomes between patients with FEP and CHR-P suggests that they may represent qualitatively different groups of help-seekers. Individuals with FEP form a heterogeneous group of patients with psychosis, among whom previous psychosocial and educational development and disorganization symptoms are decisive clinical factors predicting functional outcomes. Help-seekers with CHR-P represent individuals suffering from long-term affective disorders with mostly temporary distress that increases psychotic-like symptoms. In both groups of patients, resolving positive symptoms correlated with a functional improvement, while the continuation of affective (depression and anxiety) symptoms seemed to prevent further improvement in functioning. In patients with CHR-N, subclinical positive symptoms are mild, and functional recovery is minor. In all three patient groups, the ability to work at the time of the first visit for treatment was considerably reduced and greatly predicted their future functioning.

## Conclusions and implications

At the end of follow-up, the functional outcomes of patients with FEP improved considerably to the same level as in patients with CHR-N, whose functioning did not improve significantly. More than 90% of patients with FEP received antipsychotic medication, which probably improved the lower functioning of patients with FEP related to positive psychotic symptoms.

In addition to poor premorbid adjustment and disorganization symptomatology, poor education and work situations were associated with poor functional outcomes in patients with clinical or subclinical psychotic symptoms. Thus, psychosocial interventions aiming to improve studying and working abilities are needed to improve the future functioning of patients with severe psychotic and nonpsychotic disorders [95]. Support for education and employment considerably improve school participation and the ability to work of patients with FEP [96], and individual placement and support improve the ability to work and succeed in school, leading to competitive employment compared with traditional vocational rehabilitation [97]. Neurocognitive remediation combined with supported employment may further improve the working ability of patients with a severe mental illness [98–100]. Interventions aimed at treating depression-anxiety syndrome may improve functional outcomes, particularly in patients with CHR-P.

## Supplementary Information

**Additional file 1.**

**Additional file 2: Supplementary Table 1**. Repeated bivariate measure ANOVA of functional outcomes for sociodemographic characteristics by diagnostic groups. Significant *p* values bolded.

**Additional file 3: Supplementary Table 2**. Repeated bivariate ANOVA of functional outcomes for baseline symptoms (SIPS), childhood adverse and trauma experiences (TADS).

**Additional file 4: Supplementary Table 3**. Repeated bivariate measures ANOVA of functional outcomes for neuropsychological tests by diagnostic groups. Significant associations bolded.

**Additional file 5: Supplementary Table 4a**. Repeated measures ANOVA of functional outcome for patients with first-episode psychosis (FEP, *n* = 105). **Supplementary Table 4b**. Repeated measures ANOVA of functional outcomes for patients with confirmed clinical high-risk to psychosis (CHR-P, *n* = 49). **Supplementary Table 4c**. Repeated measures ANOVA of functional outcome for patients with nonconfirmed clinical high-risk to psychosis (CHR-N, *n* = 40).

## Data Availability

The TEPS dataset is not available for ethical reasons. The study information provided to the participants and the informed consent form they have signed includes a statement that the data collected will be used only for this particular study, not for other purposes.
