# Supplementary material for: Short-term functional outcome in psychotic patients: results of the Turku early psychosis study (TEPS)
Source: BMC Psychiatry. 2021 Dec 2;21:602. doi: 10.1186/s12888-021-03516-4 (PMC8641211; doi:10.1186/s12888-021-03516-4)
Supplement: Supplementary file 1 — Additional file 1. [file 12888_2021_3516_MOESM1_ESM.docx]

**Short-term functional outcome in psychotic patients: Results of the Turku Early Psychosis Study (TEPS).**

Raimo K. R. Salokangas^1^, Tiina From^1^, Tuula Ilonen^1^, Sinikka Luutonen^1^, Markus Heinimaa^1^, Reetta-Liina Armio^1^, Heikki Laurikainen^1^, Maija Walta^1^, Janina Paju^1^, Anna Toivonen^1^, Päivi Jalo^1,2^, Lauri Tuominen^1,3^, Jarmo Hietala^1,2^.

Author affiliations

^1^ Department of Psychiatry, University of Turku, Turku Finland

^2^ Department of Psychiatry, Turku University Hospital, Turku Finland

# ^3^ [Royal Ottawa Mental Health Centre](https://www.researchgate.net/institution/Royal_Ottawa_Mental_Health_Centre), Ottawa, Canada

Corresponding author

Raimo K. R. Salokangas

Department of Psychiatry

Kunnallissairaalantie 20, FIN-20700

Turku, Finland.

*E-mail address:* Raimo.K.R.Salokangas@utu.fi

**Supplementary Material: Aims and Materials and Methods**

**Turku Early Psychosis Study (TEPS)**

TEPS is a comprehensive investigation of patients with clinical and subclinical psychotic symptoms and healthy controls (HCs) using standardized clinical and neuropsychological assessments and self-reported instruments, as well as neuroimaging and laboratory investigations.

The investigations were carried out in accordance with the latest version of the Declaration of Helsinki. The study design and protocols were approved by the ethics committee of the Turku University Hospital (Approval numbers TEPS ETMK 64/180/2011, PRONIA ETMK 99/180/2013 and METSY ETMK 98/180/2013). Written informed consent was obtained from participants after the procedure had been fully explained.

The patient sample comprises patients with first-episode psychosis (FEP), patients with the SIPS/SOPS criteria confirmed clinical high-risk of psychosis (CHR-P) and patients who had subclinical psychotic symptoms not fulfilling the SIPS/SOPS criteria and designated nonconfirmed clinical high-risk of psychosis (CHR-N).

1. Aims

TEPS has practical and etiological aims.

A. Practical aims:

1. To investigate the psychiatric, somatic and functional states of patients with FEP, CHR-P and CHR-N and compare them with HCs.
2. To investigate the treatment profiles of the patients with FEP, CHR-P and CHR-N before and after participating in the study.
3. To investigate the clinical and functional course of the study patients at one-, two- and five-year follow-up visits and compare their outcomes with previous outcome studies.
4. To compare neurocognitive profiles between the FEP, CHR-P, CHR-N and HC groups and to investigate the associations of patients’ neurocognitive profiles with their clinical and functional outcomes.
5. To compare outcomes and actualization of treatment between participating and nonparticipating patients with FEP, CHR-P, and CHR-N.
6. To make suggestions for improving the examination of patients with FEP, CHR-P and CHR-N, planning and executing their treatment.

B. Etiological aims

1. To investigate structural and connective differences between patients with FEP and CHR-P based on structural (sMRI and DTI) and functional (fMRI) neuroimaging findings and their associations with neurocognitive deficits and the use of neuroleptic drugs.
2. To study differences in neurotransmitters (dopamine, 5-HT and cannabinoids) between patients with FEP and CHR-P and their associations with the use of neuroleptics.
3. To study metabolomics factors in patients with FEP and CHR-P and their association with health behaviors and the use of neuroleptics.

2. Sample

The study patients were recruited in two phases between October 2011 and December 2017 (the first phase started October 15, 2011, and the second phase started March 21, 2014) from mental health services of the Turku University Hospital District in Finland. The majority of the patients were recruited from two mental hospitals (n=182) and psychiatric outpatient clinics (n=101). Additional patients with CHR (n=19) were recruited by psychiatric nurses in Turku primary care. In addition, healthy controls (n=121) were recruited from the general population (n=73) and from Turku University of Applied Sciences (n=48).

The general criteria for study participants were an age of 18 to 50 years, adequate skills in Finnish and the first admission or contact to psychiatric services (hospital or psychiatric outpatient clinic). FEP was defined based on the DSM-IV criteria and included schizophrenia, delusional and bipolar psychoses, acute transient psychoses and other psychoses. CHR-P was defined based on the ultrahigh-risk criteria: Attenuated Psychotic Symptoms (APS), Brief Limited Psychotic Symptoms (BLIPS), and Genetic risk and reduction of function assessed using the 3.0/5.0 version of the Structured Interview for Prodromal Syndromes (SIPS/SOPS), including Global Assessment of Functioning (GAF) [1]. Exclusion criteria for study patients were a previous psychotic disorder and IQ < 70.

Patient recruitment was carried out as described below. When an eligible patient attended psychiatric services for the first time, the personnel completed a screen to determine whether the patient was possibly psychotic or at high risk of psychosis. The personnel asked if the patient was preliminarily willing to participate in the TEPS, which was explained to the patient. The willing patients expressed their preliminary willingness by providing written consent. In primary care settings, psychiatric nurses completed a short PROD screen questionnaire [2] with a written description of a patient’s symptoms. The study group assessed the completed screens and invited the patients who possibly fulfilled the inclusion criteria to participate in the study examinations. During the first interview, the TEPS was explained to the patient, and written consent was obtained. After diagnostic assessments, the patient was categorized as a patient with FEP or CHR-P. The patient who was at high risk of psychosis according to health care personnel but did not fulfil the SIPS/SOPS criteria were patients with an unconfirmed clinical high risk of psychosis (CHR-N).

HCs (n=121) were recruited in two stages. First, blocks of ten HC candidates of the same age and gender as the study patients were extracted from the population register of the Turku University Hospital District and contacted first by letter and a few weeks later by phone until the first willing participant in each block was identified. Those individuals who were willing to participate in the study were interviewed by phone to ensure that they fulfilled the HC inclusion criteria. If so, they were invited for interviews and other examinations. Additional HCs were recruited from the Turku University of Applied Sciences by an announcement to obtain a sufficient number of younger HCs. A description of the TEPS was mailed to the students who were preliminarily willing to participate, and then they were contacted by phone to ensure that they fulfilled the HC inclusion criteria. Otherwise, their examinations followed the same protocol as those of the population HCs.

The exclusion criteria for HCs were a history of a clinical psychiatric disorder (DSM-IV Axis I disorder) and somatic illness requiring treatment (cardiovascular, kidney, liver, blood, gastrointestinal, pulmonary, metabolic, hormonal and neurological illnesses, continuous medication, alcohol or drug dependence, and history of head trauma with unconsciousness or other brain damage) and contraindications for MRI. Additional exclusion criteria for the HCs in the second phase (n=67) included no psychosis or major affective disorder among first-degree relatives. The final TEPS sample comprises 130 patients with FEP, 61 patients with CHR-P, 49 patients with CHR-N and 121 HCs. The recruitment process is described in the chart flow below (Figure 1).


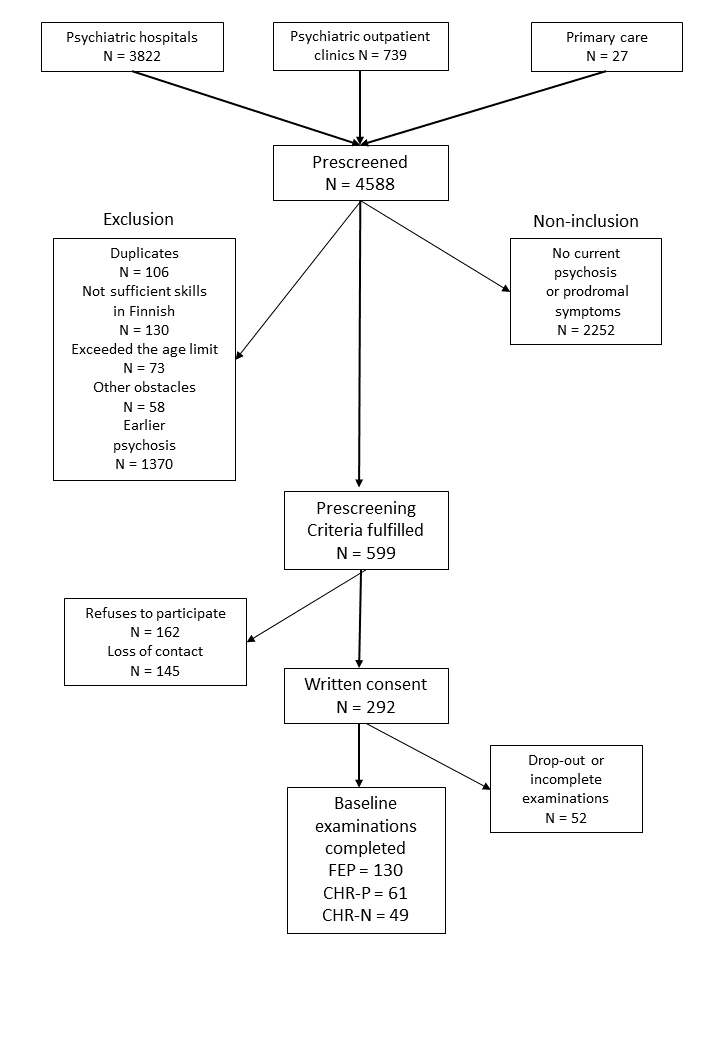


Figure 1. Chart flow for recruitment of TEPS patients.

3. Methods

3.1 Baseline examinations and follow-up instruments used in study patients and healthy controls

The study patients were extensively examined at baseline and follow-up. The list of information received and instruments used at various stages of the study are provided in Table 1.

3.2. Self-report schedules at baseline and follow-up

At the first interview, the study subjects received two sets of self-reporting questionnaires, which they returned at the second interview. At follow-up visits, the study patients had completed selected self-report schedules. All self-report schedules are listed in Tables 3.

3.3. Neuropsychological tests for all TEPS subjects

Neuropsychological tests were not performed before the patients with psychosis recovered from their manifest psychosis, usually 3 to 4 weeks after admission to a hospital ward. The neuropsychological tests used in the TEPS are listed in Table 3.

3.2. Laboratory tests

In addition to basic laboratory tests, measurements of the levels of fasting glucose, lipids, liver enzymes (GT and ALAT), thyroid tests (T4V and TSH), creatinine and EKG were recorded for all TEPS subjects at T0 (123 patients with FEP, 52 patients with CHR-P, 42 patients with CHR-N, and 106 HCs) and at T1 (51 patients with FEP, 22 patients with CHR-P, 18 patients with CHR-N, and 62 HCs). Additionally, separate tubes containing blood samples were treated and frozen for immunological, genetic, metabolic and GWAS analyses.

3.3. Neuroimaging

All scans were performed at the Turku PET Centre with a Philips Ingenuity TF 3-Tesla PET/MR scanner. The following sequences were scanned: T1-weighted (ultrafast gradient echo 3D, TR = 8.1 ms, TE = 3.7 ms, flip angle 7°, FOV = 256 × 256 × 176 mm^3^ and voxel size 1 × 1 × 1 mm^3^), T2-weighted (turbo spin echo 3D, TR = 2500 ms, TE = 309 ms, flip angle 90°, FOV = 256 × 256 × 176 mm^3^ and voxel size 1 × 1 × 1 mm^3^) and 63-directional diffusion tensor sequences (echo planar imaging, TR = 9500 ms, TE = 120 ms, flip angle 90°, FOV = 256 × 256 × 160 mm^3^ and voxel size 2 × 2 × 2 mm^3^).

Three hundred ten study subjects (102 patients with FEP, 57 patients with CHR-P, 39 patients with CHR-N, and 112 HCs) were scanned at baseline, and 139 study subjects (42 patients with FEP, 22 patients with CHR-P, 16 patients with CHR-N, and 59 HCs) were scanned at T1. Structural T1-wieghted and T2-wieghted sequences are used to measure morphometric parameters of white or gray matter, such as volume, shape, surface area or thickness. DTI is a method that measures the white matter architecture and integrity in the brain. DTI relies on the diffusion of water molecules.

The volume of the lateral nucleus of the amygdala is affected in patients with CHR-P and FEP. In the early course of the psychotic illness, a reduction in the volume of the basal nucleus was observed. Furthermore, among patients with FEP, greater childhood maltreatment experiences are associated with a smaller lateral nucleus of the amygdala, supporting a role for environmental stressors in altering the morphology of this structure during the development of psychosis [3].

To date, amygdalar subnuclei and hippocampal subregion volumes have been analyzed using either T1-wieghted or both T1-wieghted and T2-wieghted sequences. Further analyses with DTI and cortical thickness are in progress.

3.4. Follow-up examinations

All study subjects who were examined at baseline were invited to participate in follow-up studies when 9 months elapsed from the baseline examination. The study subjects who were recruited in the second phase were also examined when 18 months had elapsed from the baseline examination. The results of follow-up examinations are listed in Tables 1, 2 and 3.

3.5. Case note examination and telephone interviews

Medical case notes (patient records) of all TEPS patients were scrutinized (RKRS), and the GAF score, SOFAS score, occurrence of psychotic (yes/no), depressive (yes/no) and anxiety (yes/no) symptoms of patients who did not attend follow-up examinations were recorded at the follow-up time points T1 and T2. The evaluations of GAF and SOFAS scores and occurrence of psychiatric symptoms were based on the information recorded during the visits closest to the T1 and T2 time points. Additionally, patients and/or their relatives and/or their doctors, with the patient’s permission, were contacted and interviewed by phone (RKRS). In telephone interviews, patients’ GAF and SOFAS scores and the occurrence of psychiatric symptoms were estimated in relation to the follow-up time points. When no or limited information was available from case notes (most often treatment was ended), study participants’ functioning (GAF and SOFAS scores) was based on information related to their living situation, housing, interpersonal relationships, working, psychiatric symptoms and treatment.

The functional status (GAF and SOFAS scores) and the occurrence of psychotic, depression and anxiety symptoms were available for 237 (T0), 236 (T1) and 235 (T2) patients. Based on information received during follow-up interviews and from the patient’s case notes, the transition to psychosis was detected [4].

Table 1. Interview examinations and instruments used at the baseline (T0) and follow-up examinations (T1, T2).

| TEPS - Observer-rating questionnaires | T0 | | T1 | | T2 |
| --- | --- | --- | --- | --- | --- |
|  |  |  |  |  |  |
|  | Phase 1 | Phase 2 | Phase 1 | Phase 2 | Phase 2 |
| Structured Interview for Prodromal Syndromes (SIPS) 3.0 [1] | x |  | x |  |  |
| Premorbid Adjustment Scale (PAS) [5] | x | x |  |  |  |
| Structured Clinical Interview for DSM IV (SCID I) [6] | x | x |  | x | x |
| Strauss and Carpenter Prognostic Scale [7] | x |  | x |  |  |
| Scale for the Assessment of Negative Symptoms (SANS) [8,9] | x | x | x | x | x |
| Simpson-Agnus Scale (SAS) - short version [10] | x | x | x | x |  |
| Anosognosia | x | x | x | x |  |
| Treatment Compliance | x | x | x | x |  |
| Social and Occupational Functioning Assessment Scale (SOFAS) [11] | x | x | x | x |  |
| Global Assessment of Functioning (GAF) [11] | x | x | x | x | x |
| Grip on Life and Goals of Life (GRIP) [12] | x | x | x | x |  |
| The Brief Psychiatric Rating Scale (BPRS) [13] | x |  | x |  |  |
| Structured Interview for Prodromal Syndromes (SIPS) 5.0 [1] |  | x |  | x | x |
| Comprehensive Assessment of the At-Risk Mental States (CAARMS) [14] |  | x |  | x | x |
| Functional Remission in General Schizophrenia (FROGS) [15] |  | x |  | x | x |
| Schizophrenia Proneness Instrument (SPI-A: Cognitive disturbances (COGDIS) / Cognitive-Perceptual disturbances (COPER)) [16] |  | x |  | x | x |
| Global Functioning: Social/Role (GF:S/R) [17] |  | x |  | x | x |
| Positive And Negative Symptom Scale (PANSS) [18] |  | x |  | x | x |

Table 2. Self-rating instruments at baseline (T0) and follow-ups (T1, T2).

| TEPS - Self-rating questionnaires | T0 | | T1 | | T2 |
| --- | --- | --- | --- | --- | --- |
|  |  |  |  |  |  |
|  | Phase 1 | Phase 2 | Phase 1 | Phase 2 | Phase 2 |
| Childhood Adversities [19] | x | x |  |  |  |
| Lifetime Stressful Experiences [20] | x | x | x | x |  |
| Social Support Scale Revised (confidants) [21] | x | x | x | x |  |
| Leisure-time Physical Activity [22] | x | x | x | x |  |
| Health Behavior [23] | x | x | x | x |  |
| 15D: A 15-dimensional Measure of HRQOL [24] | x | x | x | x |  |
| Mood Disorder Questionnaire (MDQ [25]) | x | x | x | x |  |
| Self-rated Physical Fitnes [26] |  |  |  |  |  |
| Beck Anxiety Inventory (BAI) [27] | x | x | x | x |  |
| Every Day Experiences by the Obsessive-Compulsive Inventory – Revised (OCI-R) [28] | x | x | x | x |  |
| Sense of Mastery [29] | x | x | x | x |  |
| Depression Scale (DEPS) [30,31] | x | x | x | x |  |
| Visual Analogues of Attitude of Others [32] |  |  |  |  |  |
| Life Satisfaction [32] | x | x | x | x |  |
| The Personality Diagnostic Questionnaire for DSM-IV (PDQ-4) [33-35] | x |  |  |  |  |
| The Schizotypal Personality Questionnaire - Brief (SPQ-B) [36] | x |  |  |  |  |
| Dissociative Experiences Scale (DES) [37,38] | x |  |  |  |  |
| Self-reported Smoking Status [39] | x | x | x | x |  |
| The Fagerström Test for Nicotine Dependence (FTND) [40] | x | x | x | x |  |
| Trauma and Distress Scale (TADS) [41,42] | x | x |  |  |  |
| Level of Expressed Emotion Scale (LEE) [43] | x | x | x | x | x |
| Beck Depression Inventory I (BDI) [44] | x |  | x |  |  |
| Alcohol Use Disorders Identification Test (AUDIT) [45] | x | x | x | x |  |
| Questionnaire on Self-reported Use of Any Illicit Drug [46] | x | x | x | x |  |
| Edinburgh Handedness Inventory (EHI) [47] | x | x |  |  |  |
| Beck Depression Inventory II (BDI) [48] |  | x |  | x | x |
| Coping Inventory for Stressful Situations – 24 items (CISS-24) [49] |  | x |  | x | x |
| Childhood Trauma Questionnaire (CTQ) [50] |  | x |  |  |  |
| Everyday Discrimination Scale – Modified Version (EDS) [51] |  | x |  | x | x |
| The Multidimensional Scale for Perceived Social Support (MSPSS) [52] |  | x |  | x | x |
| NEO Five Factor Inventory of Personality Traits (NEO-FFI) [53] |  | x |  |  |  |
| Resilience Scale for Adults (RSA) [54] |  | x |  | x | x |
| Social Phobia Inventory (SPIN) [55] |  | x |  | x | x |
| WHO Quality of Life Questionnaire – Brief Version (WHO-QOL-BREF) [56] |  | x |  | x | x |
| Bullying Scale (BSc) [57] |  | x |  | x | x |
| Wisconsin Schizotypy Scales [58] |  | x |  |  |  |

Table 3. Neuropsychological examinations at baseline (T0) and at one-year follow-up (T1).

| TEPS - Neurocognition | Citation | T0 | | T1 | |
| --- | --- | --- | --- | --- | --- |
|  |  |  |  |  |  |
|  |  | Phase 1 | Phase 2 | Phase 1 | Phase 2 |
| Trail Making Test A [59] | Reitan 1992 | x | x |  | x |
| Trail Making Test B[59] | Reitan 1992 | x | x |  | x |
| Digit Symbol (WAIS III) [60] | Wechsler, 1997/2005 | x | x |  | x |
| Verbal Fluency Phonetic [61,62] | Blair & Spreen, 1989, Spreen and Strauss 1998 | x | x |  | x |
| Verbal Fluency Semantic [61,62] | Blair & Spreen, 1989, Spreen and Strauss 1998 | x | x |  | x |
| Spatial Span subtest of the Wechsler Memory Scale-III [63] | Wechsler, 1997/2008 | x | x |  | x |
| Letter-Number Span (WAIS-III) (LNS) [60] | Wechsler, 1997/2005 | x | x |  | x |
| Hopkins Verbal Learning Test - Revised (HVLT-R) [64] | Brandt & Benedict, 2001 | x | x |  | x |
| Brief Visuospatial Memory Test - Revised (BVMT-R) [65] | Benedict 1997 | x | x |  | x |
| Neuropsychological Assessment Battery (NAB), Mazes [66] | White & Stern 2003 | x | x |  | x |
| Wechsler Adult Intelligence Scale (WAIS III) Vocabulary [60] | Wechsler, 1997/2005 | x | x |  |  |
| Mayer-Salovey-Caruso Emotional Intelligence Test (MSCEIT): Managing emotions [67,68] | Mayer et al. 2002, Eack et al. 2010 | x | x |  |  |
| Wisconsin Card Sorting Test (WCST) [69] | Heaton et al. 1993 | x | x |  |  |
| Rorschach Comprehensive System (RCS) [70] | Mihura et al. 2013 | x | x |  | x |
| Wechsler Adult Intelligence Scale (WAIS III) Matrices [60] | Wechsler 1997/2005 |  | x |  |  |
| Wechsler Adult Intelligence Scale (WAIS III) Information [60] | Wechsler 1997/2005 |  | x |  |  |
| Continuous Performance Task (CPT-IP) [71] | Brambilla et al. 2007 |  | x |  | x |
| Self-Ordered-Pointing Task (SOPT) [72] | Ross et al. 2007 |  | x |  | x |
| Hinting Task and Interpretation of Visual Jokes [73,74] | Thompson et al. 2012, Bertrand et al. 2007 |  | x |  | x |
| Vocabulary Test [75] | Fuller et al. 2002 |  | x |  | x |

Refences

1. McGlashan TH, Miller TJ, Woods SW et al. Structured interview for prodromal syndromes. Version 3.0 and 5.0. Unpublished manual. Connecticut, New Haven: Yale School of Medicine, PRIME Research Clinic; 2001.

2. Heinimaa M, Salokangas RKR, Ristkari T et al. PROD-screen – a screen for prodromal symptoms of psychosis. Int J Meth Psych Res. 2003;12:92-104.

3. Armio RL, Laurikainen H, Ilonen T, et al. Amygdala subnucleus volumes in psychosis high-risk state and first-episode psychosis. Schizophr Res. 2020;215:284-292. doi: 10.1016/j.schres.2019.10.014. Epub 2019 Nov 16. PMID: 31744752

4. Ruhrmann S, Schultze-Lutter F, Salokangas RK, et al. Prediction of psychosis in adolescents and young adults at high risk: results from the prospective European prediction of psychosis study. Arch Gen Psychiatry. 2010;67(3):241-51.

5. Cannon-Spoor H, Potkin G, Wyatt J. Measurement of premorbid adjustment in chronic schizophrenia. Schizophr Bull. 1982;8:470–484.

6. First MB, R. L. Spitzer, M. Gibbon, J. B. W. Williams. Structured Clinical Interview for DSM-IV-TR Axis I Disorders, Research Version, Patient Edition. (SCID-I/P). Biometrics Research, New York State Psychiatric Institute, New York; 2002.

7. Strauss JS, Carpenter WT Jr. The prognostic scale. Schizophr Bull 1977;3:209–213.

8. Andreasen NC. The Scale for the Assessment of Negative Symptoms (SANS). Iowa: University of Iowa; 1983.

9. Andreasen NC. The Scale for the Assessment of Negative Symptoms (SANS): conceptual and theoretical foundations. Br. J. Psychiatry Suppl. 1989;155:49–58.

10. Simpson GM, Angus JWS. A rating scale for extrapyramidal side effects. Acta Psychiatr Scand Suppl. 1970;212:11–19.

11. Goldman HH, Skodol AE, Lave TR. Revising axis V for DSM-IV: a review of measures of social functioning, Am. J. Psychiatry 1992;149:1148–1156.

12. Salokangas RKR, Räkköläinen V, Alanen Y. Maintenance of grip on life and goals of life: a valuable criterion for evaluating outcome in schizophrenia. Acta Psychiatr Scand. 1989;80:187-193.

13. Overall JE, Gorham RG. The Brief Psychiatric Rating Scale. Psychological Reports. 1962;10:799–812.

14. Yung AR, Yuen HP, McGorry PD, et al. Mapping the onset of psychosis: the Comprehensive Assessment of At-Risk Mental States. Aust N Z J Psychiatry. 2005;39(11-12):964-971. doi: 10.1080/j.1440-1614.2005.01714.x. PMID: 16343296.

15. Llorca PM, Lançon C, Lancrenon S et al. The “Functional Remission of General Schizophrenia” (FROGS) scale: Development and validation of a new questionnaire. Schizophr Res. 2009;113:218–225.

16. Schultze-Lutter F, Addington J, Ruhrmann S, Klosterkotter J. Schizophrenia Proneness Instrument, Adult Version (SPI-A), 2007

17. Cornblatt BA, Auther AM, Niendam T et al. Preliminary Findings for Two New Measures of Social and Role Functioning in the Prodromal Phase of Schizophrenia. Schizophr Bull. 2007;33:688–702.

18. Kay S, Fiszbein A, Opler LA. The Positive and Negative Syndrome Scale (PANSS) for schizophrenia. Schizophr Bull. 1987;13:261-376.

19. Pirkola SP, Isometsa ̈E, Suvisaari J, et al. DSM-IV mood-, anxiety- and alcohol usedisorders and their comorbidity in the Finnish general population. Results from the Health2000 Study. Soc Psychiatry. Psychiatr Epidemiol. 2005;40:1–10.

20. Korkeila K, Korkeila J, Vahtera J, Kivimäki M, Kivelä SL, Sillanmäki L, Koskenvuo M. Childhood adversities, adult risk factors and depressiveness - A poplution study. Social Psychiatry and Psychiatr Epidemiol. 2005;40(9):700-706. https://doi.org/10.1007/s00127-005-0969-x

21. Blumenthal JA, Burg MM, Barefoot J, Wlliams RB, Haney T, Zimet G. Social support, type A behavior, and coronary artery disease. Psychosom Med. 1987:331–340. doi: 10.1097/00006842-198707000-00002.

22. Barengo NC, Antikainen R, Borodulin K, Harald K, Jousilahti P. Leisure-Time Physical Activity Reduces Total and Cardiovascular Mortality and Cardiovascular Disease Incidence in Older Adults. J Am Geriatr Soc. 2017;65(3):504–510.

23. Männistö S, Laatikainen T, Helakorpi S, Valsta LM. Monitoring diet and diet-related chronic disease risk factors in Finland. Public Health Nutr. 2010;13(6A):907–914.

24. Sintonen H. The 15D instrument of health-related quality of life: properties and applications. Ann Med. 2001;33:328–336.

25. Hirschfeld RMA, Williams JBW, Spizer RL et al. Development and validation of a screening instrument for bipolar spectrum disorder: The Mood Disorder Questionnaire. Am J Psychiatry. 2000;157:1873-1875

26. Solomon A, Borodulin K, Ngandu T, Kivipelto M, Laatikainen T, Kulmala J. Self-rated physical fitness and estimated maximal oxygen uptake in relation to all-cause and cause-specific self-rated physical fitness mortality. Scand J Med Sci Sport. 2018;28(2):532–540.

27. Beck AT, Epstein N, Brown G, Steer RA. An inventory for measuring clinical anxiety: psychometric properties. J Consult Clin Psychol. 1988;56:893–897.

28. Foa EB, Huppert JD, Leiberg S et al. The Obsessive-Compulsive Inventory: development and validation of a short version. Psychol Assess. 2002;14(4):485-496.

29. Pearlin LI, Morton A, Menagham EG, Lieberman MA, Muilan JT. The Stress Process. J Health Soc Behav. 1981;22:337-356

30. Salokangas RKR, Stengård E, Poutanen O: DEPS - Uusi väline depression seulontaan. (DEPS - A new tool for screening depression; in Finnish.) Duodecim 1994;110:1141-1148.

31. Salokangas RKR, Poutanen O, Stengård E. Screening for depression in primary care. Development and validation of the Depression Scale, a screening instrument for depression. Acta Psychiatr Scand. 1995;92:10-16.

32. Salokangas RKR, From T, Luutonen S, Hietala J. Adverse childhood experiences leads to perceived negative attitude of others and the effect of adverse childhood experiences on depression in adulthood is mediated via negative attitude of others. Eur Psychiatry 2018;54:27-34. doi: 10.1016/j.eurpsy.2018.06.011. Epub 2018 Jul 21.

33. Hyler SE, Rieder RO. Personality Diagnostic Questionnaire - revised. New York: Psychiatric Institute New York State; 1987.

34. Hyler SE, Skodol AE, Kellman HD, et al. Validity of the Personality Diagnostic Questionnaire – revised: comparison with two structured interviews. Am J Psychiatry. 1990;147:1043–1048.

35. Hyler SE, Skodol AE, Kellman HD, et al. Validity of the Personality Diagnostic Questionnaire – revised: a replication in an outpatient sample. Compr Psychiatry. 1992;33;73–77.

36. Raine A. The SPQ: A scale for the assessment of schizotypal personality based on DSM-III-R criteria. Schizophr Bull. 1991;17:556–564.

37. Carlson EB, Putnam FW, Ross CA et al. Validity of the Dissociative Experiences Scale in screening for multiple personality disorder: a multicenter study. Am J Psychiatry. 1993;150(7):1030-1036.

38. Carlson EB, Putnam FW. An update on the Dissociative Experiences Scale. Dissociation. 1993;6(1):16–27 (Finnish translation: Tanskanen A. (1997) Dissociative Experiences Scale, DES II. Finnish version. http://www.sidran.org/store/index.cfm?fuseaction=product.display&Product_ID=62 17.7.2008).

39. Kestilä L, Koskinen S, Martelin T, et al. Influence of parental education, childhood adversities, and current living conditions on daily smoking in early adulthood. Eur J Public Health. 2006;16(6):617–626.

40. Heatherton TF, Kozlowski LT, Frecker RC, Fagerstrom KO. 1991, The Fagerström Test for Nicotine Dependence: a revision of the Fagerstrom Tolerance Questionnaire. Br J Addict. 1991;86:1119-1127.

41. Patterson P, Skeate A, Schultze-Lutter F, et al. TADS-EPOS 1.2. Unpublished manual. University of Birmingham; 2002.

42. Salokangas RKR, Schultze-Lutter F, Patterson P, et al. Psychometric properties of the Trauma and Distress Scale, TADS,in an adult community sample in Finland. Eur J Psychotraumatol. 2016;7:30062

43. Cole JD, Kazarian SS. The Level of Expressed Emotion Scale: a new measure of expressed emotion. J Clin Psychol. 1988;44:392–397.

44. Beck AT, Ward CH, Mendelson M, Mock J, Erbaugh, J. An inventory for measuring depression. Arch Gen Psychiatry. 1961;4:561-571

45. Saunders JB, Aasland OG, Babor TF, De La Fuente, Grant M. Development of the Alcohol Use Disorders Identification Test (AUDIT): WHO Collaborative Project on Early Detection of Persons with Harmful Alcohol Consumption II. Addiction 1993;88:791-804.

46. Latvala A, Tuulio-Henriksson A, Perälä J, et al. Prevalence and correlates of alcohol and other substance use disorders in young adulthood: A population-based study. BMC Psychiatry. 2009;9:73.

47. Veale JF. Edinburgh Handedness Inventory—Short Form: A revised version based on confirmatory factor analysis. Laterality: Asymmetries of Body, Brain Cogn. 2014;19:164–177.

48. Beck AT, Steer RA, Ball R, Ranieri W. Comparison of Beck Depression Inventories -IA and -II in psychiatric outpatients. J Pers Assess. 1996;67:588–597.

49. Endler NS. D. Parker, D. T. de Ridder, G. L. van Heck, CISS: Coping inventory for stressful situations. Harcourt; 2004.

50. Bernstein D, Fink L. Childhood Trauma Questionnaire: A retrospective self-report manual. San Antonio: The Psychological Cooperation;, 1998.

51. Williams DR, Yu Y, Jackson JS, Anderson NB. Racial Differences in Physical and Mental Health: Socio-economic Status, Stress and Discrimination. J Health Psychol. 1997;2:335–351.

52. Zimet GD, Powell SS, Farley GK, Werkman S, Berkoff KA. Psychometric Characteristics of the Multidimensional Scale of Perceived Social Support. J Pers Assess 1990;55:610–617.

53. Costa P, McCrae R. Revised NEO personality inventory (NEO PI-R) and NEO five-factor inventory (NEO-FFI): Professional manual. Psychological Assessment Resources, Incorporated, 1992.

54. Friborg O, Hjemdal O, Rosenvinge J, Martinussen M. A new rating scale for adult resilience: what are the central protective resources behind healthy adjustment? Int J Methods Psychiatr Res. 2003;12:65–76.

55. Connor KM, Davidson JRT, Churchill LE et al. Psychometric properties of the Social Phobia Inventory (SPIN): New self-rating scale. Br J Psychiatry. 2000;176, 379–386.

56. World Health Organisation. WHOQOL-BREF: introduction, administration, scoring and generic version of the assessment: field trial version. Geneva, Switzerland, 1996.

57. Haidl T, Schneider N, Dickmann K, et al on behalf of the PRONIA-consortium. Validation of the Bullying Scale for Adults -Results of the PRONIA-study. J Psychiatr Res. 2020 Oct;129:88-97. doi: 10.1016/j.jpsychires.2020.04.004..

58. Winterstein BP, Ackerman TA, Silvia PJ, Kwapil TR. Psychometric properties of the Wisconsin Schizotypy Scales in an undergraduate sample: Classical test theory, item response theory, and differential item functioning. J Psychopathol Behav Assess. 2011:33(4), 480–490.

59. Reitan RM. TMT, Trail Making Test A & B, 1992.

60. Wechsler D. Wechsler Adult Intelligence Scale – 3rd Edition.: San Antonio, TX: Psychological Coorperation; 1997.

61. Blair J, Spreen O. Predicting premorbid IQ: A revision of the National Adult Reading Test. The Clin Neuropsychol. 1989;3:129-136

62. Spreen O, Strauss E. A compendium of neuropsychological tests. Oxford: Oxford University Press; 1998.

63. Wechsler D. Wechsler Adult Intelligence Scale – III, Cleveland, Ohio: The Psychological Corporation. Finnish translation. Psykologien Kustannus Oy, Helsinki, Finland 2005 and Wechsler D. Wechsler Memory Scale (3rd ed.) The Psychological Corporation, Harcourt Brace Jovanovich, New York. Finnish translation. Psykologien Kustannus Oy, Helsinki, Finland 2008.

64. Brandt J, Benedict RHB. Hopkins verbal learning test – Revised. Administration manual. Lutz, FL: Psychological Assessment Resources; 2001.

65. Benedict RHB. Brief visuospatial memory test - revised: Professional manual. Lutz, FL: Psychological Assessment Resources, Inc; 1997.

66. White T, Stern RA. Neuropsychological Assessment Battery (NAB): Demographically Corrected Norms Manual. Lutz, FL: Psychological Assessment Resources, Inc; 2003.

67. Mayer JD, Salovey P, Caruso D. MSCEIT technical manual. Toronto, Canada: Multi-Health Systems; 2002.

68. Eack SM, Greeno CG, Pogue-Geileet MF et al. Assessing social-cognitive deficits in schizophrenia with the Mayer-Salovey-Caruso Emotional Intelligence Test. Schizophr Bull. 2010;36.370–380.

69. Heaton RK, Chelune GJ, Talley JL, Kay GG, Curtiss G. Wisconsin Card Sorting Test Manual: Revised and Expanded. Odessa, FL: Psychological Assessment Resources; 1993.

70. Mihura J, Meyer G, Dumitrascu N, Bombel G. The Validity of Individual Rorschach Variables: Systematic Reviews and Meta-Analyses of the Comprehensive System. Psychol Bull. 2013;139(3):548-605.

71. Brambilla P, Macdonald AV, Sassi RB et al. Context processing performance in bipolar disorder patients. Bipolar Disord. 2007;9:230–237.

72. Ross TP, Hanouskova E, Giarla K, Calhoun E, Tucker M. The reliability and validity of the self-ordered pointing task. Arch Clin Neuropsychol. 2007:22;449–458.

73. Thompson, A, Papas A, Bartholomeusz C et al. Social cognition in clinical “at risk” for psychosis and first episode psychosis populations. Schizophr Res. 2012;141:204–209.

74. Bertrand MC, Sutton H, Achim AM, Malla AK, Lepage M. Social cognitive impairments in first episode psychosis. Schizophr Res. 2007:95;124–133.

75. Fuller R, Nopoulos P, Arndt S et al. Longitudinal assessment of premorbid cognitive functioning in patients with schizophrenia through examination of standardized scholastic test performance. Am J Psychiatry. 2002;159:1183–1189.
