# Supplementary material for: Short-term functional outcome in psychotic patients: results of the Turku early psychosis study (TEPS)
Source: BMC Psychiatry. 2021 Dec 2;21:602. doi: 10.1186/s12888-021-03516-4 (PMC8641211; doi:10.1186/s12888-021-03516-4)
Supplement: Supplementary file 5 — Additional file 5: Supplementary Table 4a. Repeated measures ANOVA of functional outcome for patients with first-episode psychosis (FEP, n = 105). Supplementary Table 4b. Repeated measures ANOVA of functional outcomes for patients with confirmed clinical high-risk to psychosis (CHR-P, n = 49). Supplementary Table 4c. Repeated measures ANOVA of functional outcome for patients with nonconfirmed clinical high-risk to psychosis (CHR-N, n = 40). [file 12888_2021_3516_MOESM5_ESM.docx]

| Supplementary Table 4a. Repeated measures ANOVA of functional outcome for patients with first-episode psychosis (FEP, n=105). | | | | | |
| --- | --- | --- | --- | --- | --- |
|  |  |  |  |  |  |
| Between subjects effect | df | F | p | Par. η^2^ |  |
| Marital status | 1 | 10.91 | **0.001** | 0.09 |  |
| Basic education | 2 | 4.59 | **0.012** | 0.08 |  |
| Work situation | 3 | 5.13 | **0.002** | 0.13 |  |
| SIPS disorganized symptoms | 1 | 15.04 | **<0.001** | 0.13 |  |
| Appropriate answers (XA%) | 1 | 4.99 | **0.028** | 0.05 |  |
| Late adolescence PAS | 1 | 9.58 | **0.003** | 0.08 |  |
| Parameter estimations | B | t | p | CI95% | |
| Functioning at baseline (GAFT0) |  |  |  |  |  |
| Single | -5.07 | -1.89 | 0.062 | -10.40 | 0.25 |
| Non-single | - |  |  |  |  |
| Comprehensive school or less | 3.36 | 1.29 | 0.200 | -1.81 | 8.53 |
| High school | -1.98 | -0.57 | 0.567 | -8.81 | 4.85 |
| College | - |  |  |  |  |
| Employed | 12.46 | 3.18 | **0.002** | 4.70 | 20.23 |
| Unemployed | 10.10 | 2.27 | **0.025** | 1.28 | 18.92 |
| Sick leave | 7.21 | 1.46 | 0.148 | -2.60 | 17.02 |
| Temporary retirement | - |  |  |  |  |
| SIPS disorganized symptoms | -1.20 | -3.86 | **<0.001** | -1.81 | -0.58 |
| Appropriate answers (XA%) | 27.47 | 2.78 | **0.006** | 7.86 | 47.08 |
| Late adolescence PAS | -0.15 | -0.61 | 0.540 | -0.63 | 0.33 |
| Functioning at 9 months (GAFT1) |  |  |  |  |  |
| Single | -8.93 | -2.80 | **0.006** | -15.27 | -2.59 |
| Nonsingle | - |  |  |  |  |
| Comprehensive school or less | -3.27 | -1.05 | 0.294 | -9.43 | 2.88 |
| High school | -11.17 | -2.72 | **0.008** | -19.31 | -3.04 |
| College | - |  |  |  |  |
| Employed | 10.86 | 2.33 | **0.022** | 1.61 | 20.10 |
| Unemployed | 0.46 | 0.09 | 0.932 | -10.05 | 10.96 |
| Sick leave | 9.55 | 1.62 | 0.108 | -2.13 | 21.23 |
| Temporary retirement | - |  |  |  |  |
| SIPS disorganized symptoms | -0.70 | -1.91 | 0.058 | -1.44 | 0.03 |
| Appropriate answers (XA%) | 21.15 | 1.80 | 0.075 | -2.20 | 44.50 |
| Late adolescence PAS | -0.78 | -2.70 | **0.008** | -1.35 | -0.21 |
| Functioning at 18 months (GAFT2) |  |  |  |  |  |
| Single | -8.72 | -2.64 | **0.009** | -15.26 | -2.18 |
| Nonsingle | - |  |  |  |  |
| Comprehensive school or less | -3.68 | -1.15 | 0.254 | -10.03 | 2.68 |
| High school | -13.11 | -3.10 | **0.003** | -21.51 | -4.72 |
| College | - |  |  |  |  |
| Employed | 10.23 | 2.13 | **0.036** | 0.69 | 19.77 |
| Unemployed | -0.10 | -0.02 | 0.986 | -10.94 | 10.74 |
| Sick leave | 9.47 | 1.56 | 0.122 | -2.58 | 21.53 |
| Temporary retirement | - |  |  |  |  |
| SIPS disorganized symptoms | -1.18 | -3.09 | **0.003** | -1.93 | -0.42 |
| Appropriate answers (XA%) | 7.98 | 0.66 | 0.513 | -16.12 | 32.07 |
| Late adolescence PAS | -1.00 | -3.34 | **0.001** | -1.59 | -0.41 |

Par. η^2^ = Partial Eta Squared

| Supplementary Table 4b. Repeated measures ANOVA of functional outcomes for patients with confirmed clinical high-risk to psychosis (CHR-P, n=49). | | | | | |
| --- | --- | --- | --- | --- | --- |
|  |  |  |  |  |  |
| Between subjects effect | df | F | p | Par. η^2^ |  |
| SIPS disorganized symptoms | 1 | 7.24 | **0.010** | 0.13 |  |
| Late adolescence PAS | 1 | 6.28 | **0.016** | 0.11 |  |
| Parameter estimations | B | t | p | CI95% | |
| Functioning at baseline (GAFT0) |  |  |  |  |  |
| SIPS disorganized symptoms | -1.33 | -3.93 | **<0.001** | -2.01 | -0.650 |
| Late adolescence PAS | -0.24 | -1.12 | 0.267 | -0.66 | 0.19 |
| Functioning at 9 months (GAFT1) |  |  |  |  |  |
| SIPS disorganized symptoms | -1.85 | -3.56 | **0.001** | -2.90 | -0.81 |
| Late adolescence PAS | -0.76 | -2.33 | **0.024** | -1.42 | -0.11 |
| Functioning at 18 months (GAFT2) |  |  |  |  |  |
| SIPS disorganized symptoms | -0.04 | -0.06 | 0.953 | -1.52 | 1.44 |
| Late adolescence PAS | -0.88 | -1.91 | 0.062 | -1.8 | 0.04 |

Par. η^2^ = Partial Eta Squared

| Supplementary Table 4c. Repeated measures ANOVA of functional outcome for patients with nonconfirmed clinical high-risk to psychosis (CHR-N, n=40). | | | | | |
| --- | --- | --- | --- | --- | --- |
|  |  |  |  |  |  |
| Between subjects effect | df | F | p | Par. η^2^ |  |
| Basic education | 2 | 9.08 | **0.001** | 0.31 |  |
| Work situation | 3 | 4.40 | **0.009** | 0.25 |  |
| SIPS general symptoms | 1 | 8.41 | **0.006** | 0.17 |  |
| Nonconfirmed clinical high risk to psychosis | | | |  | |
| Parameter estimations | B | t | p | CI95% | |
| Functioning at baseline (GAFT0) |  |  |  |  |  |
| Comprehensive school or less | -3.79 | -1.46 | 0.151 | -9.03 | 1.44 |
| High school | -0.00 | -0.00 | 1.000 | -10.96 | 10.96 |
| College | - |  |  |  |  |
| Employed | 5.49 | 1.38 | 0.175 | -2.55 | 13.53 |
| Unemployed | 7.98 | 1.74 | 0.089 | -1.26 | 17.22 |
| Sick leave | 4.69 | 0.72 | 0.475 | -8.47 | 17.86 |
| Temporary retirement | - |  |  |  |  |
| SIPS general symptoms | -1.05 | -3.15 | **0.003** | -1.72 | -0.38 |
| Functioning at 9 months (GAFT1) |  |  |  |  |  |
| Comprehensive school or less | -14.25 | -3.58 | **0.001** | -22.28 | -6.21 |
| High school | -15.37 | -1.85 | 0.072 | -32.19 | 1.45 |
| College | - |  |  |  |  |
| Employed | 16.49 | 2.70 | **0.010** | 4.15 | 28.82 |
| Unemployed | 8.55 | 1.22 | 0.230 | -5.63 | 22.73 |
| Sick leave | 1.20 | 0.12 | 0.905 | -19.02 | 21.41 |
| Temporary retirement | - |  |  |  |  |
| SIPS general symptoms | -0.66 | -1.29 | 0.205 | -1.69 | 0.37 |
| Functioning at 18 months (GAFT2) |  |  |  |  |  |
| Comprehensive school or less | -16.05 | -3.98 | **<0.001** | -24.20 | -7.89 |
| High school | -17.92 | -2.12 | **0.040** | -34.98 | -0.86 |
| College | - |  |  |  |  |
| Employed | 20.93 | 3.38 | **0.002** | 8.41 | 33.44 |
| Unemployed | 13.53 | 1.90 | 0.065 | -0.86 | 27.91 |
| Sick leave | 12.44 | 1.23 | 0.227 | -8.06 | 32.95 |
| Temporary retirement | - |  |  |  |  |
| SIPS general symptoms | -1.31 | -2.53 | **0.015** | -2.36 | -0.26 |

Par. η^2^ = Partial Eta Squared
